# Supplementary material for: Multi-user conflict resolution mechanisms for smart home environments
Source: PeerJ Comput Sci. 2023 Jun 23;9:e1443. doi: 10.7717/peerj-cs.1443 (PMC10319268; doi:10.7717/peerj-cs.1443)
Supplement: Supplemental Information 2 [file peerj-cs-09-1443-s002.docx]

**APPENDIX A.1 PRE-TEST QUESTIONNAIRE**

**Participant’s Demographic Information**

**Please circle (e.g. © ) an option as appropriate for each of the following.**

(1) Please specify your age group:

a. Less than 18

b. 18 – 25

c. 26 – 35

d. 35 or above

(2) Sex:

a. Male

b. Female

(3) What is your role in the family?

a. Father

b. Mother

c. Children

(4) Highest academic qualification:

a. Less than High School

b. High School/College

c. Bachelors Degree

d. Masters Degree

e. Doctorate (PhD)

(5) Do you have any idea about smart home environment?

a. Yes

b. No

(6) Do you have experience user conflicts while you used the application in your home environment?

a. Yes

b. No

(6) Do you have experience of using smart environment especially with multi-user .environment?

a. Yes

b. No

**AFTER SCENARIO QUESTIONNAIRE**

For each of the statements below, circle the rating of your choice.

1. How satisfied are you with using the automatic resolution approach.

**Very Unsatisfied 1 2 3 4 5 6 7 Very Satisfied**

**Comments:**

2. How satisfied are you with using the mixed resolution approach.

**Very Unsatisfied 1 2 3 4 5 6 7 Very Satisfied**

**Comments:**

3. How satisfied are you with the mechanisms used in the automatic resolution approach.

**Very Inappropriate**  **1 2 3 4 5 6 7 Very Appropriate**

**Comments:**

4. Rate if the automatic resolution approach was appropriate for the scenario where it’s applied.

**Very Inappropriate**  **1 2 3 4 5 6 7 Very Appropriate**

**Comments:**

5. Rate if the mixed resolution approach was appropriate for the scenario where it’s applied.

**Very Inappropriate**  **1 2 3 4 5 6 7 Very Appropriate**

**Comments:**

6. How satisfied are you with the time the system took to complete the resolution processes.

**Very Much Time 1 2 3 4 5 6 7 Very Little Time**

**Comments:**

**POST-TEST QUESTIONNAIRE**

**Please answer the following questions based on your experience of the performed usability test of Conflict Resolution approaches.**

(1) We have implemented automatic resolution according to the priority wise (the highest priority user will be served and if two users have the same priority the user who entered first will be served) is this appropriate for the home situation.

**Very Inappropriate**  **1 2 3 4 5 6 7 Very Appropriate**

**Comments:**

(2) Please rank the following three categories of resolution approaches by numbering them (i.e. from 1 to 3) 3 is the highest and 1 is the lowest .

a. Automatic Resolution Approach: _________

b. Mediated Resolution Approach: _________

c. Mixed Resolution Approach: _________

(3) We would appreciate it, if you could give any suggestions or comments that you think will help us to improve the proposed system. Please also feel free to discuss your general feedback orally.

**Thanks for giving your valuable time and feedback**
